# Changes in Post-Acute Trajectories for Patients With Serious Mental Illness After Payment Reforms

**DOI:** 10.1093/geroni/igaf122.3556

**Published:** 2025-12-31

**Authors:** Evelyn Hart, Harsha Amaravadi, Rachel Prusynski, Amber Sabbatini, Natalie Leland, Donald Fogelberg, Tracy Mroz

**Affiliations:** University of Washington, Seattle, Washington, United States; University of Washington, Seattle, Washington, United States; University of Washington, Seattle, Washington, United States; University of Washington, Seattle, Washington, United States; University of Pittsburgh, Pittsburgh, Pennsylvania, United States; University of Washington, Seattle, Washington, United States; University of Washington School of Medicine, Seattle, Washington, United States

## Abstract

Post-acute care (PAC) services help older adults transition home after hospitalization, yet people with serious mental illness (SMI) may experience barriers to accessing PAC services from skilled nursing facilities (SNFs) and home health agencies (HHAs). We examined whether SMI as a secondary diagnosis was associated with PAC use, and whether PAC use changed following implementation of Medicare payment reforms: the Patient Driven Payment Model (PDPM) in 2019 for SNFs and the Patient Driven Groupings Model (PDGM) in 2020 for HHAs. We analyzed claims for Traditional Medicare beneficiaries aged 18+ hospitalized between 2018–2021 (n = 29,001,357), excluding those with SMI as the primary reason for hospitalization; 18% were admitted to a SNF and 16% to HHA after hospital discharge. Fixed-effects logistic regression with two-way clustered standard errors estimated odds of SNF and HHA admission (in separate models) comparing those with versus without SMI, adjusting for patient, facility, and community characteristics. Interaction terms assessed whether associations between PAC admissions and SMI differed following payment reforms. Secondary SMI was associated with higher odds of SNF admission (OR = 1.48) but lower odds of HHA admission (OR = 0.95). PDPM was linked to slightly reduced SNF use (OR = 0.98), while PDGM was associated with increased HHA use (OR = 1.10). Interaction terms indicated modest attenuation of baseline differences (SNF: OR = 1.06; HHA: OR = 1.02). All findings were statistically significant. Though both PDPM and PDGM aim to compensate facilities fairly for patient care needs, payment reform had differing effects by PAC setting, reflecting underlying divergent pathways to PAC for patients with secondary SMI.

